# Moyamoya angiopathy: radiological follow-up findings in Finnish patients

**DOI:** 10.1007/s00415-020-09837-w

**Published:** 2020-04-22

**Authors:** Marika Savolainen, Johanna Pekkola, Satu Mustanoja, Tiina Tyni, Juha Hernesniemi, Leena Kivipelto, Turgut Tatlisumak

**Affiliations:** 1 Department of Neurology, South Karelia Central Hospital, Valto Käkelän katu 1, 53130 Lappeenranta, Finland; 2 Clinical Neurosciences, Neurology, University of Helsinki and Helsinki University Hospital, Helsinki, Finland; 3 Department of Radiology, HUS Medical Imaging Center, Helsinki University Hospital and University of Helsinki, Helsinki, Finland; 4 Department of Child Neurology, Helsinki University Hospital, Helsinki, Finland; 5 Juha Hernesniemi International Center for Neurosurgery, Henan Provincial People’s Hospital, Zhengzhou, People’s Republic of China; 6 Clinical Neurosciences, Neurosurgery, University of Helsinki and Helsinki University Hospital, Helsinki, Finland; 7 Department of Clinical Neurosciences, Institute of Neuroscience and Physiology, Department of Neurology, Sahlgrenska Academy at University of Gothenburg, Sahlgrenska University Hospital, Gothenburg, Sweden

**Keywords:** Moyamoya angiopathy, Follow-up, MRI, Ivy sign, White matter lesions, Cerebral microbleeds

## Abstract

**Background and purpose:**

Moyamoya angiopathy (MMA) is a chronic progressive disorder, but imaging changes observed over time are not yet characterized in European populations. We analyzed the progression of MMA with magnetic resonance imaging and angiography (MRI and MRA) in our Finnish MMA registry. Stage classification based on MRA findings was used to evaluate the progress of the disease.

**Methods:**

32 patients with MMA were evaluated with MRI and MRA and compared to previous imaging. The follow-up imaging was done 103 (range 6–380) months after the MMA diagnosis, and 64 (range 6–270) months after the previous imaging. We graded the disease stage according to the previously described MRA grading scale.

**Results:**

No acute lesions, including silent ischemic strokes were found in the follow-up image compared to latest available previous image. One patient had an asymptomatic intracerebral hemorrhage since the last imaging. Ivy sign was observed in 22% of the patients in the follow-up image. Six percent (*n* = 2) had microhemorrhages and 9% (*n* = 3) white matter lesions in the follow-up imaging. The MRA grade was evaluated from the follow-up images and it was 3 and 2.5 points (right and left, respectively). Fifty-six percent (*n* = 18) had old ischemic lesions in the follow-up image. Majority (71%) of the old ischemic lesions were large anterior circulation infarcts.

**Conclusions:**

A slow progression of MMA-related changes on MRI/MRA was found, being in line with our previous reports suggesting a rather benign course of the disease in the Finnish population.

## Introduction

Moyamoya disease (MMD) and moyamoya syndrome (MMS), referred as the moyamoya angiopathy (MMA), is a chronic progressive steno-occlusive angiopathy at the distal portions of internal carotid arteries (ICAs) and their proximal branches, with typical collateral artery formation, the moyamoya vessels. Digital subtraction angiography (DSA) is considered the golden standard for imaging the MMA, however, the non-invasive magnetic resonance imaging (MRI) is currently commonly used for diagnostic and follow-up imaging [1]. The radiological findings associated with MMD include stenosis or occlusion of the large branches of the internal carotid artery (middle cerebral artery, MCA, and anterior cerebral artery, ACA), rarely the posterior cerebral artery (PCA), development of collaterals in the basis of the brain giving the “puff of smoke” impression, ischemic and hemorrhagic parenchymal brain lesions, ivy sign, cerebral microbleeds (CMBs), and white matter lesions (WMLs). While in general, MMA changes are irreversible and progressive, the speed and extent of the progress of the MMA changes in brain parenchyma and arterial tree in European white patient populations are not well-studied. Most of the studies concerning these radiological findings are done in Asian populations [2–5], and there is one single study by Wenz concerning CMBs in the German population [6]. We accomplished follow-up imaging with MRI and MRA in 32 Finnish MMA patients to evaluate silent/asymptomatic progression of the angiopathy, the occurrence of ivy sign, CMBs and WMLs, silent or overt infarcts and intracerebral hemorrhages, and to grade the disease stage.

## Materials and methods

This study was performed at the Departments of Neurology and Neurosurgery, Helsinki University Hospital (HUH), Helsinki, Finland. The local ethics committee approved the study (154/13/03/00/10). All MMA patients who agreed to come for an outpatient clinical follow-up visit coupled with MRI/MRA gave a written consent and were included to this study.

We searched for all diagnosed MMD and MMS patients referred to our hospital, and collected patient data into a detailed database as reported earlier [7], and called them for a face-to-face follow-up visit including head MRI and head and cervical artery MRA. Our HUH-MMA database includes detailed data on the patients’ medical history, family history of stroke and MMA, medication on admission and preventive medication at discharge, hospital admission data, clinical manifestation and time course, treatment and procedures, discharge details, laboratory tests on admission, and radiological data. Our HUH-MMA database includes patients from whole Finland, but for this study we limited the follow-up imaging to those living in HUH catchment area, with a population of 1.6 million.

### MRI and MRA

In this study, all patients underwent MRI and MRA imaging with a 3.0 T scanner (Philips Achieva, Best, the Netherlands). The imaging sequences included T1-weighed and T2-weighed 3D-sequences with isotropic acquisition in sagittal plane, axial FLAIR sequence (slice thickness 4 mm), axial diffusion-weighed sequence (slice thickness 4 mm, spacing 5 mm), axial susceptibility-weighed sequence (thickness 1 mm, spacing 0.5 mm), time-of flight (TOF) MRA of the cerebral arteries, and flow-based MRA of the cervical arteries. For the patients with extracranial-intracranial bypasses, the cerebral artery TOD MRA was performed also with gadolinium contrast agent (Dotarem 279.3 mg/ml, Guerbet, France). We used the MRA grading described by Houkin et al. to evaluate the progress of the disease [8]. Both hemispheres were evaluated separately. In the grading system ICA and MCA were graded from 0 to 3 (0 normal, 3 invisible) and ACA and PCA from 0 to 2 (0 normal, 2 invisible). The MRA score is the total points from four main cerebral arteries (minimum 0 and highest 10). The MRA score was classified into four grades (MRA score 0–1, grade 1; 2–4, grade 2; 5–7 grade 3; and 8–10, grade 4) [8]. We also evaluated the presence of ivy sign, CMBs, pattern of ischemic lesions, and presence of WMLs, and occurrence of new silent or overt ischemic or hemorrhagic lesions since previous imaging. Patients with revascularization operation done were evaluated to see if the bypasses were still patent. The analysis of the radiological data was done by a neuroradiologist (JP) together with a neurologist (MS). Ischemic lesions were divided into small (< 1.5 cm) and large anterior or posterior lesions. Extent of WMLs was classified according to the Fazekas classification [9]. The sequences of the latest previous image used in the comparison to see the progression varied because they were done in the normal clinical need settings and not systemically using the same sequences as done in the imaging performed for this study.

### Statistical analyses

Frequencies, means, and medians were calculated using the SPSS software (IBM Corp. Released 2012. IBM SPSS Statistics for Windows, Version 23.0. Armonk, NY: IBM Corp.). Crosstabs and Chi-squared test were used to compare the groups. A two-sided *p* value < 0.05 is considered significant.

## Results

32 patients (7 male, 22%) agreed to follow-up imaging (two had MMS; one with Down’s syndrome with bilateral disease and one with neurofibromatosis type 1 with unilateral disease, see Table 1 for demographic data). Seventy-three percent (22/30) of the MMD patients had bilateral disease. Ten (31%) patients had had revascularization surgery. The follow-up imaging was done 103 (range 6–380) months after the initial diagnosis of MMV and 64 (range 6–270) months after previous (baseline) imaging.

**Table 1:** Demographic data and disease type of Finnish MMV patients (*n* = 32)

	Patients
All patients *n* (%)	32 (100)
Female (%)	25 (78)
Male (%)	7 (22)
Age at the time of first symptoms, mean (range)	35 (4–62)
Age at the time of first symptoms, median	36
Age at the time of diagnosis, mean (range)	37 (5–67)
Age at the time of diagnosis, median	38.5
Age at the time of follow-up, mean (range)	43.8 (18–70)
Age at the time of follow-up, median	48
Type of disease	
MMD (%)	30 (94)
MMD bilateral (%)	22 (69)
MMS^a^ (%)	2 (6)
Surgery (%)	10 (31)
STA-MCA (unilateral *n*)	7 (2)
EDAS (unilateral *n*)	3 (2)

*EDAS* encephalo-duro-arterio-synangiosis; *MMD* moyamoya disease; *MMS* moyamoya syndrome; *ICH* intracerebral hemorrhage; *SAH* subarachnoid hemorrhage; *STA-MCA* superficial temporal artery to middle cerebral artery; *TIA* transient ischemic attack

^a^Down’s syndrome and neurofibromatosis 1 each

Ivy sign was present in 7 (22%) patients and 2 of these had had revascularization operation done. Interestingly, in two patients ivy sign was present in previous image but not in the follow-up image. The amount of ischemic strokes (*p* = 0.36), multiple ischemic strokes (*p* = 0.42), nor the location of ischemic strokes (*p* = 0.67) did not differ between ivy sign-positive and ivy sign-negative patients. Only 2 (6%) patients had CMBs, one of them being asymptomatic and the other one having had ischemic stroke as the presenting pathology at the time of diagnosis. None had CMBs in the previous image, but only 11 had had SWI/T2* sequence done in the baseline image. 91% of the patients had no WML (Fazekas 0) and the rest (three patients) had only mild WML (Fazekas 1) and one of those had had revascularization operation. Only one had WML in the previous imaging but again only 26 had had FLAIR-sequence and thus could be evaluated. Twelve (38%) patients had completely normal brain parenchyma in the follow-up image. In the Table 2 the imaging findings are summarized.

**Table 2:** Imaging findings of the previous (baseline) image and last (follow-up) MR imaging

	Previous imaging	Follow-up imaging
MRI		
Normal parenchyma	13 patients	12 patients
New ischemic lesions		0
Intracerebral hemorrhage		1 (silent)
Silent (covert) infarction		1
CMB (no of patients with SWI/T2* sequences)	0 (11/32)	2 (32/32)
WMH (no of patients with T2/FLAIR sequences)	1 mild (26/32)	3 mild (32/32)
Ivy sign (no of patients with FLAIR sequence)	3 (24/32)	7 (32/32)
MRA		
Unilateral MM	8	8

*CMB* cerebral microbleeds; *MM* moyamoya; *MRA* magnetic resonance angiography; *MRI* magnetic resonance imaging; *WML* white matter lesions

Five (16%) patients were asymptomatic at the time of diagnosis. One of those patients had CMBs, one had ivy sign, and none had WMLs in the follow-up image. All of these patients remained asymptomatic during the follow-up time. One, the only MMS patient with Down’s syndrome, had progression of stenosis in the arteries.

None had acute ischemic or hemorrhagic stroke in the follow-up image. None had new silent ischemic strokes compared to previous imaging. Fifty-six percent (*n* = 18) of patients had old ischemic lesions, 38% had cortical lesion, and 41% had subcortical lesions in the follow-up image. Most of the old ischemic lesions located in MCA (17%), border zone (28%), or both of previous (33%) territories. Forty-four percent of the patients had multiple ischemic lesions in the follow-up image. Seventy-one percent of the ischemic lesions were large anterior circulation lesions, 6% were large posterior and the rest were small lesions in the follow-up image. Fifty-three percent of the patients had bilateral lesions in the follow-up image. None of the 8 patients with unilateral MMD had progressed to bilateral MMD during the mean follow-up period of 71 (range 12–161) months.

Only one patient had a new vascular event in the follow-up image since the last imaging. Initially this patient sought medical help because of tinnitus at the age of 39. The first brain MRI was normal, although the MRA disclosed a bilateral MMD. In the follow-up MRI performed 9 years later there were signs of old subcortical parenchymal hemorrhage on the right. However, she had not experienced neurological symptoms.

Ten patients had had revascularization operations, 3 (1 bilateral, 2 unilateral) of them had had encephalo-duro-arterio-synangiosis operation and the rest (5 bilateral, 2 unilateral) superficial temporal artery to middle cerebral artery (STA-MCA) bypass. We evaluated 10 hemispheres of those who had had STA-MCA operation and only 1 of 10 of the bypasses was not patent.

Median MRA grade was 3 and 2.5 (right and left, respectively), median scores were 6 and 4.5 points (right and left, respectively, mean 4.9 ± 2.5, 4.3 ± 2.7) in 28 patients with evaluable MRA data.

## Discussion

Our results in 32 MMA patients with a mean follow-up of 64 months between the two MRI/MRA imaging time points suggest a fairly slow progress of MMA in Finnish patients, adding new insights to our previous observations of a low frequency of clinical events in the same patient registry [10]. In line with few clinical events, new ischemic or hemorrhagic lesions were found in one patient only, and all the unilateral cases remained unilateral. Only one new vascular event was found, the sign of an old subcortical parenchymal hemorrhage. Ivy sign was observed in 22%, CMBs in 6%, and WMLs in 9% of the patients.

There is a lack of studies reporting regular clinical and imaging follow-up of MMA patients in the white populations, describing disease progress over long time. This leads to a serious shortcoming in understanding MMA disease dynamics. MMA per se may be multifactorial and heterogeneous group of different syndromes presenting with a unifying vasculopathic appearance and thus the vascular changes may progress with different speeds in different patient subgroups, individuals, or ethnic groups depending on factors we are yet unaware of. Therefore, it is useful to screen a consecutive and large-enough patient population with follow-up imaging studies, along with clinical evaluations, for describing the disease progress speed (i.e. natural course of the disease) as well as to look into various subgroups whether disease progress differs in certain subpopulations. This may further help in identifying subgroups of patients that need close monitoring and perhaps more aggressive treatments and ignites further research interest deciphering underlying unique mechanisms. Currently, there are two guidelines for MMA, one Japanese and one French. The French guideline suggests that MRI and MRA imaging should be done on a case-by-case basis according to the clinical and radiological evolution of the patient, but at least once a year during the first years [11]. Japanese guidelines do not deliver a clear follow-up approach [1]. However, the lack of broad data on the disease progression, and lack of evidence-based strategies to react to imaging-based vascular or parenchymal changes, make it difficult to give long-term follow-up plan perspectives. Therefore, more reports on large MMV patient populations with clinical and imaging-based follow-up data are necessary for developing guidelines for clinical use. According to our results, it does not seem necessary to arrange frequent routine imaging follow-up studies in clinically stable white European patients.

There are limited data comparing the imaging findings and clinically overt symptoms and signs. In a Japanese patient population with asymptomatic MMD 3/34 non-surgically treated patients experienced silent radiological changes, including cerebral infarction, CMB, and one progression of the disease stage on follow-up MRA in the 43.7 months follow-up period [12]. In another Japanese study silent CMBs were found in 2 of 20 asymptomatic patients during the 48.8 months follow-up time [13]. The number of asymptomatic patients in our study was small (*n* = 5) and their radiological findings were few, one CMB and one ivy sign.

Ishikawa described occurrence of CMBs in MMD [14]. Incidence of CMBs is found to be high, especially in the hemorrhagic onset-type MMD, and meta-analysis indicated that they may be an important factor for hemorrhagic stroke risk [4]. CMBs can be detected using T2*-weighted imaging (T2*WI) and/or susceptibility-weighted imaging (SWI). In Asian populations CMBs have been reported in 28.2–51.9% of the patients [2–4]. In a German population based study CMBs were found in 12.9% of their patient population (*n* = 101) [6] which is close to our 6% result and it seems that the incidence of CMBs is lower in European populations. Another German population based study found no CMBs after STA-MCA bypass surgery during a mean follow-up of 38.2 months [15]. Unfortunately blood-sensitive MRI sequences have not been widely used in earlier MRI imaging sessions and therefore long-time follow-up data are not extensively available on this aspect.

Reported frequency of progression from unilateral to bilateral varies from 12 to 39% over 1–15 years of follow-up periods [16–22]. In our patient population none of the unilateral cases progressed to bilateral during a mean follow-up of 71 months which could mean that the unilateral disease remains mainly unilateral or the progression to bilateral disease is an extremely slow process in most cases.

**Fig. 1:**
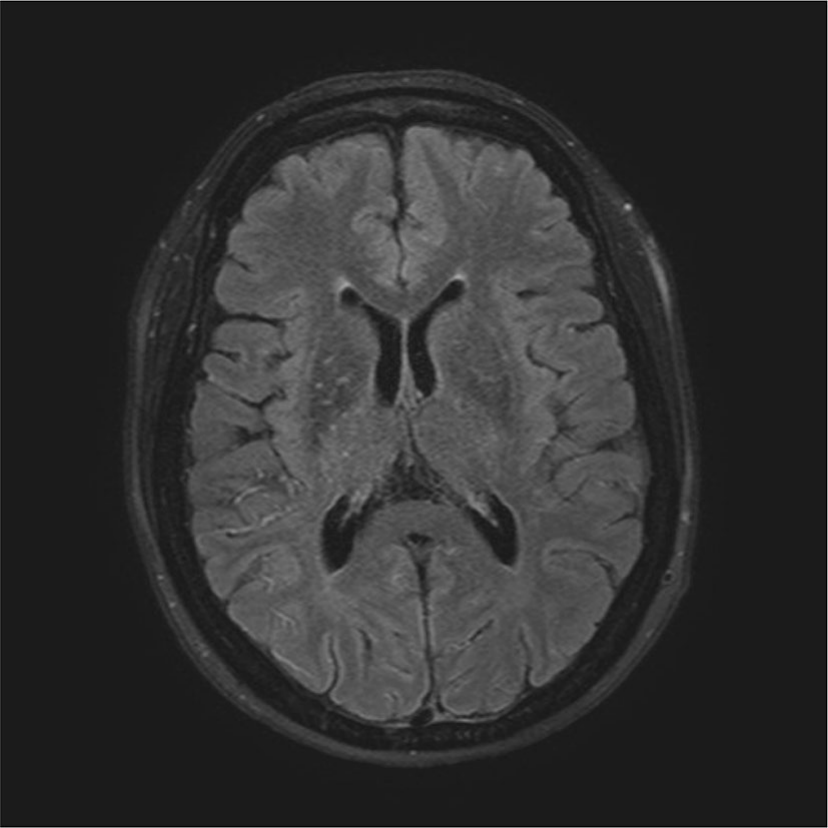
Ivy-sign in MRI

Ivy sign refers to the appearance of the brain on post-contrast T1-weighted images or FLAIR images where prominent leptomeningeal collaterals with slow blood flow and profuse contrast enhancement appear as if the brain is covered with ivy (Fig. 1) [23]. Oh et al. found ivy sign in 8 out of 12 patients (67%) [5]. Seo et al. found ivy sign in all of their 83 patients’ population [18]. In our population, ivy sign was present in 22% of the patients. Mori et al. found that the degree of the ivy sign showed a significant positive relationship with the severity of the ischemic symptoms and concluded that the ivy sign indicates decreased cerebral vascular reserve in MMD [24]. In a recent study by Kronenburg et al. they found that ivy sign was not related to the presence or absence of collaterals on DSA, nor did it reflect absent cerebrovascular reactivity [25]. Our ivy-positive patients did not differ from our ivy sign-negative patients in terms of other imaging parameters or clinical characteristics. The pathophysiology of ivy sign and its clinical significance is so far unresolved. While interpretation of images on the presence and severity of ivy sign may differ between centers, it still seems that ivy sign is far less usual in white patients compared to Asian MMD patients.

MMD patients (*n* = 21) harbored more WMLs than controls with the symptomatic side of the brain being more affected and suggested that WMLs might precede transient ischemic attacks (TIAs) [26]. Same study showed that WML volume decreased after revascularization surgery [26]. In our study, only 3 patients (9%) had WMLs and only one of them had had revascularization surgery. In the previous imaging only one had WMLs, but again unfortunately only 26/32 of the patients had FLAIR-sequence done. In a Japanese population WMLs were present in 57/100 hemispheres [27]. Our patients were slightly older that the patients in these two studies, therefore, the differences in the presence and extent of WMLs are not explained by a younger population in our study. Again, it appears that the Finnish MMA patients have substantially less WML than Asian patients.

Our study has several limitations. Unfortunately we could not include all our HUH-MMA database patients because of long distances in our country and logistical difficulties of arranging radiological standardized follow-up leaving us with a smaller number of patients who lived nearby. The imaging time points were not standardized and included a range of 6–270 months from the previous last available MRI/MRA or a range of 6–380 months from the initial MMA diagnosis. Because only the latter imaging was done with same sequences to all patients, comparing the findings of previous and latest images could not be done in all cases. The strength of our study is well investigated patients all of Finnish origin (Caucasian) and living in Finland with a homogenous patient population. Because there are only limited data on follow-up imaging changes in MMA, our study, adds new data to the field.

In conclusion, during a mean 64 month follow-up imaging study, we detected fewer ivy sign, CMBs and WMLs than previously reported in MMD patients and detected only slight deteriorations which support our previous finding of relatively benign course of MMA in our Finnish patient population.
